# Proteomics Reveal the Inhibitory Mechanism of Levodopa Against Esophageal Squamous Cell Carcinoma

**DOI:** 10.3389/fphar.2020.568459

**Published:** 2020-09-25

**Authors:** Zhenzhen Li, Xin Li, Xinyu He, Xuechao Jia, Xiaofan Zhang, Bingbing Lu, Jimin Zhao, Jing Lu, Lexia Chen, Ziming Dong, Kangdong Liu, Zigang Dong

**Affiliations:** ^1^ Department of Pathophysiology, School of Basic Medical Sciences, AMS, College of Medicine, Zhengzhou University, Zhengzhou, China; ^2^ China-US (Henan) Hormel Cancer Institute, Zhengzhou, China; ^3^ Henan Provincial Cooperative Innovation Center for Cancer Chemoprevention, Zhengzhou, China; ^4^ State Key Laboratory of Esophageal Cancer Prevention and Treatment, Zhengzhou, China; ^5^ Cancer Chemoprevention International Collaboration Laboratory, Zhengzhou, China

**Keywords:** esophageal squamous cell carcinoma, levodopa, proteomic, mitochondria, succinate dehydrogenase subunit D

## Abstract

High recurrence rates and poor survival of patients with esophageal squamous cell carcinoma (ESCC) after treatment make ongoing research on chemoprevention drugs for ESCC particularly important. In this study, we screened a large number of FDA-approved drugs and found levodopa, a drug used to treat Parkinson’s disease, had an inhibitory effect on the growth of ESCC cells. To elucidate the molecular mechanisms involved, we applied quantitative proteomics to investigate the anti-tumor activity of levodopa on ESCC. The results suggest that levodopa could down-regulate oxidative phosphorylation, non-alcoholic fatty liver disease, and Parkinson’s disease pathways. Major mitochondrial respiratory compounds were involved in the pathways, including succinate dehydrogenase subunit D, NADH-ubiquinone oxidoreductase Fe-S protein 4, and mitochondrial cytochrome c oxidase subunit 3. Down-regulation of these proteins was associated with mitochondrial dysfunction. Western blotting and immunofluorescence results confirmed the proteomics findings. Cell viability assays indicated mitochondrial activity was suppressed after levodopa treatment. Reduced mitochondrial membrane potential was detected using JC-1 staining and TMRE assays. Transmission electron microscopy revealed changes in the morphology of mitochondria. Taken together, these results indicate that levodopa inhibited the growth of ESCC through restraining mitochondria function.

## Introduction

Esophageal cancer (EC) ranks seventh and sixth in global incidence and mortality, respectively, for malignant tumors (Bray et al., 2018) with esophageal squamous cell carcinoma (ESCC) being the main histological form (He et al., 2017; Song et al., 2018; Xie et al., 2019). According to recent epidemiological investigations, the recurrence rates after middle and late stage treatment of patients with ESCC are high, and the five-year survival rate is less than 20%, (Lin et al., 2018; Liu et al., 2019; Xie et al., 2019). Therefore, it is of particularly urgency to identify compounds that may prevent ESCC recurrence and to elucidate their underlying molecular mechanisms of action. Recently, a great deal of research has focused on the new practice of using Food and Drug Administration (FDA)-approved drugs beyond their original applications. For instance, in addition to its hypoglycemic effects, metformin can also repress the progression of prostate, ovarian, and pancreatic cancers (Chen et al., 2017; Liu et al., 2018; Xu et al., 2018). Similarly, while aspirin has antipyretic and analgesic effects and is used in preventing cardiovascular disease, it may also help prevent colorectal cancer by normalizing the expression of epidermal growth factor receptor (EGFR) (Li et al., 2015). These examples support the feasibility of finding chemoprevention compounds among FDA-approved drugs. Based on this concept, we screened FDA-approved drugs and found that levodopa could effectively inhibit the growth of ESCC cells.

Levodopa is a natural dopamine biosynthesis precursor and a normal intermediate of human metabolism. It naturally exists in many plants, including as broad beans, cat beans, and mucuna (Kang et al., 2013; LeWitt, 2015). Levodopa is the most effective and commonly used drug for the treatment of Parkinson’s disease (Jones et al., 1978). It enters the brain through the blood-brain barrier and is converted to dopamine, which plays a role in the treatment of Parkinson’s disease (Abbott, 2010). Currently, proteomics are widely used in screening for biomarkers of malignant tumors and prenatal diagnosis and in research regarding the mechanism of action of drugs (Kolialexi et al., 2009; Kisluk et al., 2014; Lao et al., 2014; Dias et al., 2016; Huang et al., 2017). In the current study, levodopa was found to also dramatically restrain the growth of ESCC cells. To investigate the inhibitory mechanism of levodopa on ESCC, we performed mass spectrometry-based proteomics analysis. The results revealed that oxidative phosphorylation, non-alcoholic fatty liver disease (NAFLD), and Parkinson’s disease pathways were downregulated following levodopa treatment. Proteins such as NADH-ubiquinone oxidoreductase Fe-S protein 4 (NDUFS4), succinate dehydrogenase subunit D (SDHD), and mitochondrial cytochrome c oxidase subunit 3 (MT-CO3) were involved in these pathways and with changes related to mitochondrial dysfunction. We also demonstrated that levodopa could affect mitochondrial morphology and suppress mitochondrial activity in ESCC cells. Overall, we showed that levodopa retarded the growth of ESCC cells by restricting mitochondrial function.

## Materials and Methods

### Cell Culture

Human ESCC cell lines KYSE150 and KYSE450 were purchased from the Chinese Academy of Sciences Cell Bank (Shanghai, China). KYSE150 cells were cultured in RPMI-1640 medium (Biological Industries) supplemented with 10% fetal bovine serum (FBS) and 1% (v/v) penicillin/streptomycin solution. KYSE450 cells were cultured in Dulbecco’s Modified Eagle Medium (DMEM; Biological Industries) supplemented with 10% FBS and 1% (v/v) penicillin/streptomycin solution. All cell cultures were incubated at 37℃ in a humidified incubator with 5% CO_2_.

### Cell Proliferation Assays

KYSE150 and KYSE450 cells were seeded into 96-well plates at 3 × 10^3^ and 5 × 10^3^ cells/well, respectively, and incubated overnight. The cells were then treated for 24, 48, 72, or 96 h with various concentrations of levodopa (≥ 98% purity; Sigma-Aldrich, Cat #59927). The concentrations of levodopa used were 0, 50, 100, 200, 400, and 600 µM. At each time point, cells were stained with 4ʹ,6-diamidino-2-phenylindole (DAPI; Solarbio) and the viable cells counted. Cell number curves were plotted for each concentration at the different time points. Each point represented the mean ± standard deviation (SD) of three biological replicates.

### Anchorage-Independent Cell Growth

To evaluate anchorage-independent cell growth, Basal Medium Eagle (BME) containing 10% FBS and 40% agar with various concentrations of levodopa (0, 25, 50, 100, 200, and 400 µM) was added to 6-well plates (3 ml/well). After solidification of the bottom BME layer, KYSE150 and KYSE450 cells (8 × 10^3^ cells/well) were suspended in a top layer containing 10% FBS, 45% agar, and the indicated concentrations of levodopa. The cells were cultured for 1 to 2 wk at 37°C in a 5% CO_2_ incubator. The number of cell clones that formed were then counted and analyzed using a high-content cell imaging analysis system. For each experimental result, three replicates for each concentration were evaluated and the experiment was performed three times.

### Sample Collection for Proteome and Western Blotting Analyses

KYSE150 cells (4.5 × 10^6^) were seeded into 15 cm dishes and treated with levodopa (0 or 600 µM) for 24 h. The collected cells and proteins were stored in at −80℃ until use.

### Sample Preparation for Proteomics Analysis

The samples were sonicated three times on ice in lysis buffer (8 M urea, 1% protease inhibitor cocktail, 3 µM TSA, 50 mM NAM, and 2 mM EDTA) using a high-intensity ultrasonic processor (Scientz). The lysate was centrifuged at 12,000 × *g* at 4˚C for 10 min and the supernatant then collected. Protein concentrations were determined using a bicinchoninic acid (BCA) kit. Dithiothreitol was added to the protein solutions to a final concentration of 5 mM and then reduced for 30 min at 56˚C. Iodoacetamide (11 mM) was used to alkylate the proteins to for 15 min at room temperature in darkness. Urea concentrations of the samples were diluted to < 2 M by with 100 mM NH_4_HCO_3_. Trypsin was added at a trypsin-to-protein mass ratio of 1:50 for a first digestion overnight and then at a ratio of 1:100 for a second 4-h digestion.

### High-Performance Liquid Chromatography (HPLC) Fractionation

The tryptic peptides were fractionated using high pH reverse-phase HPLC with an Agilent 300 Extend C18 column (5 μm particles, 4.6 mm inner diameter, 250 mm length). The peptides were separated into 60 fractions using a gradient of 8% to 32% acetonitrile (pH 9.0) over 60 min. The peptides were then combined into six fractions and dried by vacuum centrifugation.

### Liquid Chromatography Tandem Mass Spectrometry (LC-MS/MS) Analysis

The peptides were dissolved in solvent A (0.1% formic acid and 2% acetonitrile aqueous solution), and then separated using an ultra-performance liquid chromatography (UPLC) system (EASY-nLC 1000; Thermo Scientific). The gradient consisted of an increase from 6% to 22% solvent B (0.1% formic acid in 90% acetonitrile) over 40 min, 22% to 35% solvent B over 12 min, 35% to 80% solvent B over 4 min, and rising to 80% solvent B for the final 4 min. A constant flow rate of 700 nl/min was used. The separated peptides were then ionized using a nanospray ionization (NSI) ion source and then analyzed using Orbitrap Fusion™ (Thermo Scientific) mass spectrometry. The electrospray voltage applied was 2.0 kV. Both the peptide parent ions and their secondary fragments were detected and analyzed using high-resolution Orbitrap. The m/z scan range was 350 to 1550 for the full scan and intact peptides were detected in the Orbitrap at a resolution of 60,000. The secondary mass spectrometer scan range was fixed at a starting point of 100 m/z and the resolution was set at 15,000. After the full scan, the first 20 peptides with the highest signal intensities were selected for entry into the higher energy collisional dissociation (HCD) pool and fragmented using 35% of the fragmentation energy. Secondary mass spectrometry was also performed. To improve the effective utilization of mass spectrometry, the automatic gain control (AGC) was set at 5E4, the signal threshold was set to 5000 ions/s, the maximum injection time was set at 200 ms, and the dynamic exclusion time for tandem mass spectrometry scans was set at 30 s to avoid repeated scans of the parent ions.

### Database Search

The tandem MS/MS data were indexed using MaxQuant search engine (v.1.5.2.8). Tandem mass spectra were searched against the SwissProt Human database concatenated with a reverse decoy database and common pollution database. Trypsin/P was specified as the cleavage enzyme, allowing for up to 2 missing cleavages. The minimum peptide length was set at 7 amino acid residues and the maximum number of peptide modifications was set at 5. The mass tolerance for precursor ions was set at 20 parts per million (ppm) in the first search and 5 ppm in the main search The mass tolerance for fragment ions was set as 0.02 Da. Alkylation on Cys was specified as a fixed modification while oxidation of Met and N-terminal acetylation were specified as variable modifications. The false discovery rate (FDR) was adjusted to < 1%.

### Data Processing Using Gene Set Enrichment Analysis (GSEA)

Using the GSEA v4.0.3 package, protein group data sets were integrated with gene Set enrichment analysis (GSEA) to analyze differences in protein expression between the treatment group and the control group. Mitochondrial protein complex gene set (GO:0098798) and respiratory electron transport gene set (R-HSA-611105) were selected for enrichment. Through previous functional annotations or experiments, GSEA determined the relevant gene sets, and sequence the genes according to the degree of differential expression of the two types of samples. Gene set enrichment analysis detected gene set expression changes and optimizes the results using P values and false discovery rate (FDR) method. The biological pathway with P value <0.01 and FDR <0.25 was considered statistically significant in the GSEA analysis. Statistical analysis and graphical plotting were conducted utilizing GSEA Software (v4.0.3).

### Western Blot Analysis

For Western blot analysis, KYSE150 cells (4.5 × 10^6^) were seeded into 15-cm dishes and treated with levodopa (0 or 600 μM) for 24 h. Cells were washed twice with cold phosphate-buffered saline (PBS). Cells were scraped from the plates in a modified radioimmunoprecipitation assay (RIPA) buffer containing 50 mM Tris HCl (pH 7.4), 1% NP-40 (v/v), 1.25% SOD (w/v), 150 mM NaCl, 1 mM EDTA•Na_2_, and 0.1% sodium dodecyl sulfate (SDS; w/v) supplemented with protease inhibitor (100×), 100 mM Na_3_VO_4_, 0.5 μM NaF, and 100 mM phenylmethanesulfonyl fluoride (PMSF). Cells were collected and placed on ice for 30 min followed by centrifugation at 14,000 rpm for 30 min at 4°C. Protein concentrations were determined using a BCA kit (Beyotime). Protein lysates loaded into a 50 μg system and separated using 12% SDS-polyacrylamide gel electrophoresis (PAGE). After separation, the proteins were transferred to polyvinylidene difluoride (PVDF) membranes (Millipore). The membranes were blocked with 5% skim milk for 1 h at room temperature and then washed four times with Tris-buffered saline. The membranes were incubated with specific primary antibody at 4°C for 16 to 18 h. Secondary antibody was then added to the membranes and incubated at room temperature for 2 h. The membranes were scanned using an Odyssey System (LI-COR, USA).

### Immunofluorescence

KYSE150 cells were seeded into 12-well plates at 1.5 × 10^5^ cells/well and incubated overnight. The cells were then treated with levodopa (0 or 600 µM) for 24 h, washed with cold PBS three times, fixed with 4% paraformaldehyde, and placed at −20°C overnight. Cells were permeabilized with 0.5% TritonX-100 solution for 10 min and blocked with 1% bovine serum albumin-PBS-Tween 20 (BSA-PBST) for 1.5 h at room temperature. The cells were then incubated with primary antibodies at 4°C overnight. Cells were subsequently incubated with fluorescent secondary antibody for 1.5 h at room temperature in darkness. Nuclei were stained using DAPI. Images were collected using a confocal microscope and attached camera.

### XTT Cell Viability Assays

KYSE150 and KYSE450 cells were seeded into 96-well plates at 3 × 10^3^ and 5 × 10^3^ cells/well, respectively, and incubated overnight. Cells were then treated for either 24 or 48 h with various concentrations of levodopa (0, 50, 100, 200, 400, and 600 µM). At each time point, 50 µl XTT detection solution (Cell Signaling Technology, cat #9095) was added to each well and incubated for another 4 h. Absorbance at 450 nm was measured using a Multiskan Sky spectrophotometer (Thermo Fisher).

### Mitochondrial Membrane Potential Detection Using JC-1 Staining

KYSE150 cells were seeded into 12-well plates at 3 × 10^4^ and 5 × 10^4^ cells/well, respectively, and incubated overnight. Cells were then treated with levodopa (0 or 600 µM) for 24 h. At each time point, cells in the positive control group were treated with 10 µM carbonyl cyanide 3-chlorophenylhydrazone (CCCP) for 20 min. All cells were then treated with JC-1 dye for 20 min and the nuclei stained with Hoechst 33342 for 30 min. Cells were immediately observed under a fluorescence microscope and photographed.

### Mitochondrial Membrane Potential Detection Using Tetramethylrhodamine Ethyl Ester (TMRE)

KYSE150 cells were seeded into 12-well plates at 3 × 10^4^ and 5 × 10^4^ cells/well, respectively, and incubated overnight. The cells were then treated with levodopa (0 or 600 µM) for 24 h. At each time point, cells in the positive control group were treated with 10 µM carbonyl cyanide-p-trifluoromethoxyphenylhydrazone (FCCP) for 10 min. Subsequently, all cells were treated with TMRE for 30 min and the nuclei stained with Hoechst 33342 for 30 min. Cells were immediately observed under a fluorescence microscope and photographed.

### Sample Preparation for Transmission Electron Microscope (TEM) Evaluation

KYSE150 and SHEE cells (4.5 × 10^6^ cells/dish) were seeded into 15-cm dishes and treated with levodopa (0 or 600 μM) for 24 h. The old media were removed and discarded and 30 ml PBS added to each of the 15 cm dishes. The cells were then digested by the addition of 3 ml Trypsin-EDTA. Cells were transferred to 50-ml tubes and centrifuged at 900 rpm for 5 min at 4°C. Supernatants were discarded and the cells fixed in 2.5% glutaraldehyde within 1 min and moved to 1.5-ml tubes to be centrifuged at 900 rpm for 5 min at 4°C. Again, the supernatants were discarded and the pelleted cells were then placed in 2.5% glutaraldehyde solution on ice and sent for TEM evaluation.

### Statistical Analysis

SPSS 17.0 software was used for all statistical analyses. The quantitative data are expressed as mean values ± SD. Significant differences were compared using the Student’s t-test or one-way analysis of variance (ANOVA). *P* < 0.05 was considered to be statistically significant.

## Results

### Levodopa Inhibited the Growth of ESCC at a Cellular Level

**Figure 1:**
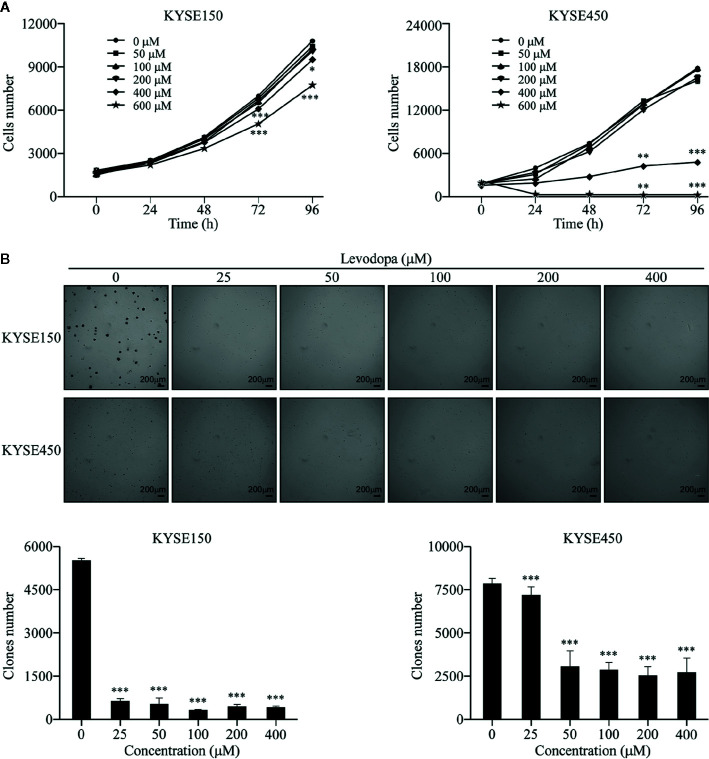
Levodopa inhibits the growth of ESCC cells. **(A)** KYSE150 and KYSE450 cells were treated with levodopa (0, 50, 100, 200, 400, and 600 μM) for 0, 24, 48, 72, and 96 h and evaluated using DAPI staining. **(B)** KYSE150 and KYSE450 cells were treated with levodopa (0, 25, 50, 100, 200, and 400 μM) for 2 wk. The asterisks indicate a significant decreases in proliferation and clone formation ability compared to that of the control (*P < 0.05, **P < 0.01, and ***P < 0.001). Values are expressed as mean ± SD (n = 3). ANOVA testing was performed; ***P < 0.001 vs. other group.

We first investigated whether levodopa could affect the growth of ESCC cells. KYSE150 and KYSE450 cells were treated with different concentrations of levodopa for various lengths of time and the number of cells then detected using DAPI staining. Results showed that levodopa could significantly inhibit the proliferation of ESCC cells (**Figure 1A**). In addition, results from the anchorage-independent assays revealed that levodopa could dramatically reduce the clone formation ability of KYSE150 and KYSE450 ESCC cells (**Figure 1B**). Taken together, these results indicate that levodopa was able to observably attenuate the growth of ESCC at a cellular level.

### Proteomics Evaluation of ESCC Cells Following Levodopa Treatment

**Figure 2:**
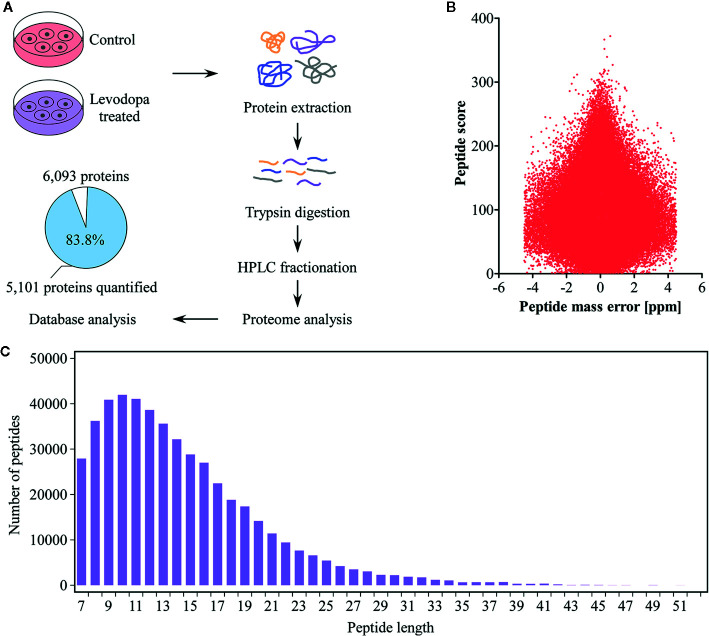
Proteomics identification of KYSE150 ESCC cells following levodopa treatment. **(A)** Experimental design of proteomic identification of KYSE150 ESCC cells after 24 h of levodopa treatment (0 or 600 μM). The percentage and number of proteins quantified are shown in the pie chart (Adj. *P* < 0.05, fold change > 1.5). **(B, C)** The distribution of peptide mass error **(B)** and peptide length **(C)** of protein peptides identified in this study. Values are expressed as mean ± SD (n = 3). ANOVA testing was performed.

To elucidate the inhibitory mechanism of levodopa against ESCC, we performed mass spectrometry-based proteome analysis of KYSE150 cells after 24 h treatment with 600 μM levodopa (**Figure 2A**). We used a label-free quantification approach to perform high-resolution LC-MS/MS analysis. A total of 6,093 proteins were identified of which 5,101 contained quantitative information. The mass error distribution of identified peptides (**Figure 2B**) was centered at 0 and concentrated in the range below 10 ppm, indicating that the mass error met the requirements. In addition, the majority of the peptides were distributed between 7 and 20 amino acid residues (**Figure 2C**), consistent with the expectations of trypsin-digested peptides. In short, the sample preparation was consistent with that of the standard.

### Global Proteomic Changes in ESCC KYSE150 Cells

**Figure 3:**
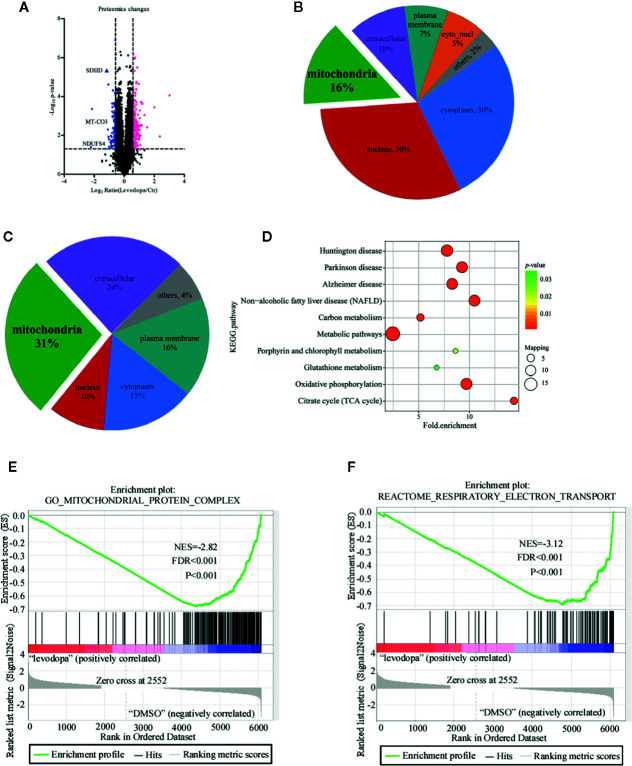
Proteomics analysis of KYSE150 ESCC cells following levodopa treatment. **(A)** A volcano plot showing the proteins that significantly changed after 24 h treatment with levodopa (600 μM). Each point represents a protein with blue indicating down-regulation and red indicating up-regulation. **(B, C)** Subcellular localization analysis of all differentially expressed proteins **(B)** and the downregulated proteins **(C)**. **(D)** A KEGG pathway enrichment map of the down-regulated proteins. Values are expressed as mean ± SD (n = 3). ANOVA testing was performed. **(E)** GSEA showed the Mitochondrial Protein Complex gene set (GO:0098798) is down regulated by levodopa treatment in KYSE150 cells. (NES, normalized enrichment score.) **(F)** GSEA showed the Respiratory electron transport gene set (R-HSA-611105) is down regulated by levodopa treatment in KYSE150 cells.

With a 1.5-fold change as the threshold and *P* < 0.05 as the standard, 245 proteins were identified as being differentially expressed of which 162 were up-regulated and 83 down-regulated (**Figure 3A**). To understand the potential role of these proteins, we performed subcellular structural localization prediction and classification analyses of the 245 differentially expressed proteins using WoLF PSORT. The results indicated that 16% of the proteins were localized in mitochondria (**Figure 3B**). Interestingly, 31% of the 83 down-regulated proteins were located in mitochondria (**Figure 3C**). Based on these findings, we speculated whether mitochondria-related proteins may play an important role in the inhibitory mechanism of levodopa. Enrichment analysis was performed using the Kyoto Encyclopedia of Genes and Genomes (KEGG) to investigate whether differentially expressed proteins had a significant enrichment trend in certain functional types. **Figure 3D** shows that following levodopa treatment (0 vs 600 μM), some pathways were down-regulated, including those related to Huntington’s disease, Parkinson’s disease, Alzheimer’s disease, NAFLD, carbon metabolism, metabolic pathways, porphyrin and chlorophyll metabolism, glutathione metabolism, oxidative phosphorylation, and the citrate cycle (TCA cycle). To further investigate the changes in the protein of the mitochondrial oxidative respiratory chain after levodopa treatment. GSEA was used to enrich the differentially expressed proteins between the treatment group and the control group. After levodopa treatment (0 and 600 μM), the differentially expressed proteins were down-regulated in Mitochondrial Protein Complex gene set (GO:0098798) (**Figure 3E**) and the Respiratory electron transport gene set (R-HSA-611105) (**Figure 3F**).

### KEGG Pathway Analysis of Proteomics Data

**Figure 4:**
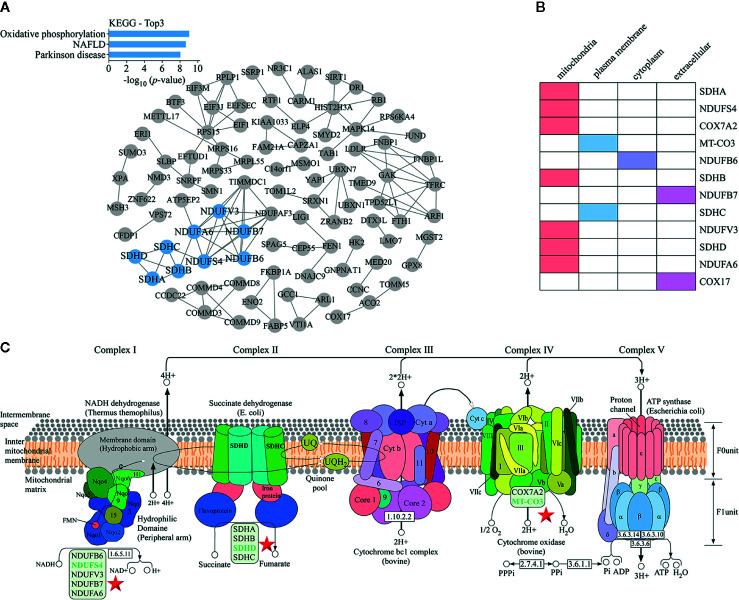
KEGG pathway analysis of proteomics data. **(A)** A protein-protein interaction network map of differentially expressed proteins. The blue nodes represent the proteins present in the top three down-regulated pathways. These proteins were enriched in the same cluster. **(B)** Statistics for the subcellular localization of proteins involved in the top three down-regulated pathways. **(C)** The oxidative phosphorylation pathway. The positions of proteins such as NDUFS4, SDHD, and MT-CO3 in the pathway are indicated with a star.

**Figure 5:**
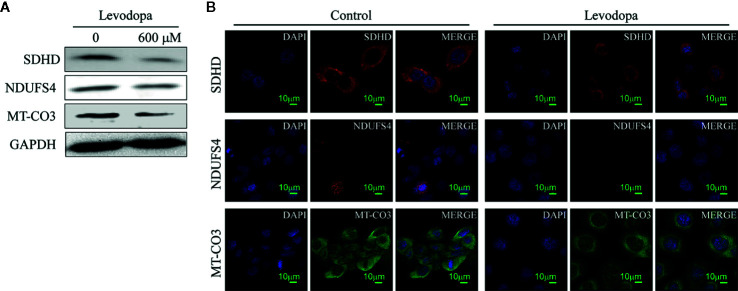
Key enzymes involved in the oxidative phosphorylation pathway were downregulated. **(A)** Western blotting results revealed the levels of SDHD, NDUFS4, and MT-CO3 decreased following levodopa treatment. **(B)** Immunofluorescence results show the downregulation of NDUFS4, SDHD, and MT-CO3 (scale, 10 μm). Values are expressed as mean ± SD (n = 3).

The protein-protein interaction network map of differentially expressed proteins (**Figure 4A**) showed that proteins involved in the top three down-regulated pathways were enriched in the same cluster. Of the 12 proteins enriched in these top three down-regulated pathways, seven were localized in mitochondria (**Figure 4B**). Mitochondria are the main sites of oxidative phosphorylation in cells. As shown in **Figure 4C**, NADH dehydrogenase 1 alpha subcomplex subunit 6 (NDUFA6), NADH dehydrogenase 1 beta subcomplex subunit 6 (NDUFB6), NADH dehydrogenase 1 beta subcomplex subunit 7 (NDUFB7), NDUFS4, and NADH dehydrogenase [ubiquinone] flavoprotein 3 (NDUFV3) were located in mitochondrial complex I; succinate dehydrogenase complex (SDH) subunit A (SDHA), SDH subunit B (SDHB), SDH subunit C (SDHC), and SDH subunit D (SDHD) were located in mitochondrial complex II; cytochrome c oxidase polypeptide 7A2 (COX7A2) and MT-CO3 were located in complex IV. Therefore, we were more focused on mitochondria-associated proteins. To verify the proteomics data, we performed Western blotting (**Figure 5A**) and immunofluorescence (**Figure 5B**). The results revealed that the protein levels of NDUFS4, SDHD, and MT-CO3 were reduced, which was consistent with the proteomics data.

### Levodopa Caused Changes in Mitochondria Function in ESCC Cells

**Figure 6:**
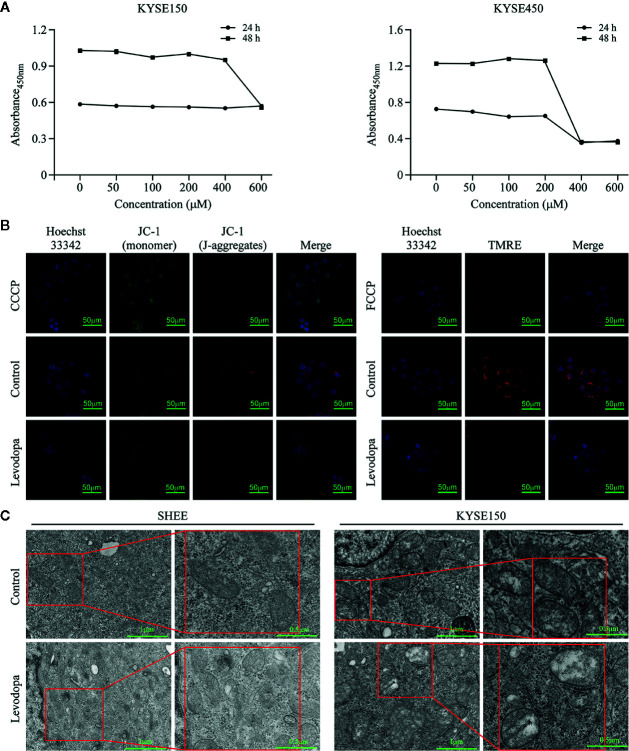
Levodopa inhibits the mitochondria function in ESCC cells. **(A)** Levodopa restrains the cellular mitochondrial dehydrogenase activity in KYSE150 ESCC cells. **(B)** Levodopa treatment decreases the mitochondrial membrane potential in KYSE150 ESCC cells as detected by JC-1 staining and TMRE assays, respectively. CCCP and FCCP were used as positive controls. **(C)** After levodopa treatment, the mitochondria of ESCC cells were swollen and the cristae sizes reduced. In contrast, the mitochondrial morphology of normal esophageal epithelial SHEE cells was not significantly changed by levodopa treatment. Values are expressed as mean ± SD (n = 3).

To investigate whether levodopa could inhibit the activity of mitochondria, we performed XTT cell viability assays. The results indicated that levodopa was able to attenuate dehydrogenase enzymes (**Figure 6A**). We also measured the mitochondrial membrane potential using JC-1 staining (**Figure 6B**) and TMRE assays (**Figure 6B**). Consistent with the expected results, the mitochondrial membrane potential decreased following levodopa treatment. To investigate whether the effect of levodopa on mitochondria function was related to morphology, we performed TEM evaluation of levodopa-treated cells. The TEM results showed that compared with that of the control group, the mitochondria of KYSE150 cells were swollen and the number of mitochondrial cristae was reduced (**Figure 6C**). In contrast, mitochondrial morphology of SHEE cells was not changed by levodopa treatment at the same concentration.

## Discussion

The high rate of recurrence in patients with ESCC results in a low five-year survival rate (Lin et al., 2018). Therefore, effective drugs are urgently needed to address this problem. FDA-approved drugs are used in clinical practice and have detailed pharmacokinetic and safety data available. Investigating new uses of these drugs can shorten development cycles, save development costs, and maximize resource utilization (Mohs and Greig, 2017). For instance, research has shown that long-term use of low-dose aspirin, which has been long approved by the FDA, has chemo-preventive effects in colorectal cancer (Li et al., 2015). This type of result unequivocally confirms the feasibility of realizing new uses for FDA-approved drugs. In the current study, we screened a large number of FDA-approved drugs and found that levodopa was able to effectively inhibit the growth of ESCC cells. Levodopa has been used clinically to treat Parkinson’s disease, a common neurodegenerative disorder; however, there has been no research regarding its use as an anti-cancer agent (Poewe et al., 2010). To elucidate the molecular mechanism of the anti-tumor activity of levodopa on ESCC, we performed a proteomics analysis of KYSE150 ESCC cells 24 h after levodopa treatment (0 and 600 μM). A total of 162 proteins were found to be up-regulated and 83 proteins down-regulated using a 1.5-fold change as the threshold and *P* < 0.05 as a standard.

KEGG enrichment analysis of differentially expressed proteins revealed that the Parkinson’s disease pathway was significantly inhibited by levodopa 24 h after treatment. This demonstrated the reliability of the proteomics data. In addition, the NAFLD pathway was also down-regulated following levodopa treatment. NAFLD is a liver manifestation of a metabolic syndrome that includes obesity and diabetes (Tian et al., 2013). NAFLD is a leading cause of liver disease worldwide and is also the leading cause of hepatocellular carcinoma (HCC) in the United States (Stickel and Hellerbrand, 2010; Younossi et al., 2016). Furthermore, through the analysis of GSEA oxidative phosphorylation was also downregulated as a result of levodopa-induced changes. Oxidative phosphorylation refers to the process of synthesizing ATP by a series of electron transfer protein complexes embedded in the inner mitochondrial membrane (van der Bliek et al., 2017). In mammalian cells, approximately 90% of ATP is derived from the oxidative phosphorylation pathway (Papa et al., 2012). It is clear that inhibition of oxidative phosphorylation in an organism will lead to abnormal energy metabolism and may also affect the growth of tumor cells.

Proteins such as NDUFS4, SDHD, and MT-CO3 are involved in these pathways. Down-regulation in the levels of these protein is related to mitochondrial dysfunction and abnormal energy metabolism. The NDUFS4 gene is located on chromosome 5q11.1 and encodes one of the subunits of mitochondrial complex I (Budde et al., 2002). Mitochondrial complex I is the first and largest enzyme in the oxidative phosphorylation system and is the core of cellular NAD^+^ recycling, accounting for approximately 40% of mitochondrial ATP production (Kuhn et al., 2015). NDUFS4 is the main entry point for electrons into the oxidative phosphorylation system (Wang et al., 2017). Studies have shown that down-regulation of NDUFS4 can result in a significant down-regulation of mitochondrial complex I activity and can block the binding of cytosol to membrane-bound proteins, thereby inhibiting the migration potential of melanoma cells (Valsecchi et al., 2013; Wang et al., 2017). In comparison, the SDHD gene is located on chromosome 11q and encodes a protein that forms one of the two transmembrane subunits of the SDH complex. It is essential for the function of the electron transport chain and mitochondrial complex II (Kugelberg et al., 2014). Studies have shown that the knockout of SDHD in human glioma SNB19 cells and human neuroblastoma SHSY5Y cells can reduce cell proliferation (Hoekstra et al., 2016). The proteomics data were confirmed by Western blotting and immunofluorescence. Our studies demonstrated that levodopa was not only able to inhibit mitochondrial activity and mitochondrial membrane potential, but also alter mitochondrial morphology in ESCC cells.

In general, levodopa inhibited the growth of ESCC cells through regulating pathways involved in oxidative phosphorylation, NAFLD, and Parkinson disease, down-regulating the levels of SDHD, NDUFS4, and MT-CO3, and inhibiting oxidative phosphorylation.

## Author's Note

The mass spectrometry proteomics data have been deposited at the ProteomeXchange Consortium (http://proteomecentral.proteomexchange.org) *via* the PRIDE partner repository with dataset identifier PXD013824. Project Name: Proteomics reveals the inhibitory mechanism of Levodopa against esophageal squamous cell carcinoma; Project accession: PXD013824.

## Data Availability Statement

The datasets presented in this study can be found in online repositories. The names of the repository/repositories and accession number(s) can be found below: http://www.proteomexchange.org/, PXD013824.

## Author Contributions

Study conception and design: ZGD, ZMD, and KL. Conducted experiments: ZL, XL, XZ, LC, and BL. Analysis and interpretation of data: XH, JZ, and JL. Drafting and critical revision of the manuscript: ZL, XJ, and KL. All authors contributed to the article and approved the submitted version.

## Funding

This work was supported by grants from the National Natural Science Foundations of China (grant numbers 81872335 and 81572812), the Natural Science Foundations of Henan (grant number 161100510300), and National Science & Technology Major Project “Key New Drug Creation and Manufacturing Program,” China (grant number 2018ZX09711002).

## Conflict of Interest

The authors declare that the research was conducted in the absence of any commercial or financial relationships that could be construed as a potential conflict of interest.
